# Could KL-6 levels in COVID-19 help to predict lung disease?

**DOI:** 10.1186/s12931-020-01560-4

**Published:** 2020-11-24

**Authors:** AN Frix, L. Schoneveld, A. Ladang, M. Henket, B. Duysinx, F. Vaillant, B. Misset, M. Moutschen, R. Louis, E. Cavalier, J. Guiot

**Affiliations:** 1 Department of Respiratory Medicine, CHU Liège, University Hospital of Liège, Domaine Universitaire du Sart-Tilman, B35, 4000 Liège, Belgium; 2 Department of Clinical Chemistry, CHU Liège, University Hospital of Liège, Domaine Universitaire du Sart-Tilman, B35, 4000 Liège, Belgium; 3 Intensive Care Unit, CHU Liège, University Hospital of Liège, Domaine Universitaire du Sart-Tilman, B35, 4000 Liège, Belgium; 4 Department of Infectious Diseases and Immunology, CHU Liège, University Hospital of Liège, Domaine Universitaire du Sart-Tilman, B35, 4000 Liège, Belgium

**Keywords:** COVID-19, Interstitial lung disease, Lung infection, Biomarker, KL-6

## Abstract

**Background:**

Coronavirus disease COVID-19 has become a public health emergency of international concern. Together with the quest for an effective treatment, the question of the post-infectious evolution of affected patients in healing process remains uncertain. Krebs von den Lungen 6 (KL-6) is a high molecular weight mucin-like glycoprotein produced by type II pneumocytes and bronchial epithelial cells. Its production is raised during epithelial lesions and cellular regeneration. In COVID-19 infection, KL-6 serum levels could therefore be of interest for diagnosis, prognosis and therapeutic response evaluation.

**Materials and methods:**

Our study retrospectively compared KL-6 levels between a cohort of 83 COVID-19 infected patients and two other groups: healthy subjects (n = 70) on one hand, and a heterogenous group of patients suffering from interstitial lung diseases (n = 31; composed of 16 IPF, 4 sarcoidosis, 11 others) on the other hand. Demographical, clinical and laboratory indexes were collected. Our study aims to compare KL-6 levels between a COVID-19 population and healthy subjects or patients suffering from interstitial lung diseases (ILDs). Ultimately, we ought to determine whether KL-6 could be a marker of disease severity and bad prognosis.

**Results:**

Our results showed that serum KL-6 levels in COVID-19 patients were increased compared to healthy subjects, but to a lesser extent than in patients suffering from ILD. Increased levels of KL-6 in COVID-19 patients were associated with a more severe lung disease.

**Discussion and conclusion:**

Our results suggest that KL-6 could be a good biomarker to assess ILD severity in COVID-19 infection. Concerning the therapeutic response prediction, more studies are necessary.

## Introduction

The rapid outbreak of coronavirus disease 2019 (COVID-19) caused by severe acute respiratory syndrome coronavirus 2 (SARS-COV-2) infection, has plainly become a public health emergency of international concern [1, 2]. Despite a wide range of clinical presentation and varying degrees of severity, more than 887.814 people worldwide have now recovered from this viral lung infection. Though the number of recoveries does provide solace, there is still little information about how these patients and their lung integrity will evolve throughout the post-infectious healing process.

Interstitial lung damage induced by viral infection conventionally leads to respiratory symptomatology associating cough, chest pain and dyspnea. Dyspnea may in some cases persist for several weeks due to lung parenchymal sequelae. Coronavirus are known for their potential to induce lung fibrosis [3].

In this context, it therefore appears imperative to identify potential biomarkers associated with alveolar pathology that could be of prognostic interest regarding the severity of lung sequelae.

Different studies highlight the importance of the quest for diagnostic or prognostic biomarkers in pulmonary fibrosing process [4–6]. Krebs von den Lungen 6 (KL-6) is a high molecular weight mucin-like glycoprotein produced by type II pneumocytes and bronchial epithelial cells. Its production is raised during epithelial lesions and cellular regeneration. In normal lungs, this glycoprotein is involved in fibroblast stimulation and apoptosis inhibition. In case of epithelial lesions, alveolo-capillary leak can occur and lead to an increase in serum KL-6 levels. Indeed, this modulation is not specific to COVID-19 infection and can be found in numerous other diseases associated with alveolar epithelial cell lesions (autoimmune diseases, radiation-associated pneumonia, drug-associated pneumonia, etc.). In COVID-19 infection, KL-6 serum levels could therefore be of interest for diagnosis, prognosis and therapeutic response evaluation.

Our study aims to compare KL-6 levels between a COVID-19 population and healthy subjects or patients suffering from interstitial lung diseases (ILDs). Ultimately, we ought to determine whether KL-6 could be a marker of disease severity and bad prognosis.

## Materials and methods

Our study retrospectively compared KL-6 levels between a cohort of 83 infected patients (COVID-19 PCR positive patients hospitalized in Liège University Hospital between March 1st to April 20th 2020) and two other groups: healthy subjects (n = 70) on one hand, and a heterogenous group of patients suffering from ILD (n = 31; composed of 16 IPF, 4 sarcoidosis, 11 others) on the other hand.

Demographical (including age, sex, past medical history), clinical (including oxygen levels, Intensive Care Unit (ICU) indication) and admission laboratory indexes (including serum CRP, serum IL-6/KL-6, serum LDH, complete blood count, renal and liver functions) were collected. Chemistry analyses were run on the Abbott Alinity platform (Abbott Park, IL, USA) and KL-6 were measured with the Fujirebio Lumipulse 1200 instrument (Tokyo, Japan). High levels of KL-6 (45.6 UI/L) where defined as the value above the mean of the normal population + 2 SD (260.42 UI/L + (2 × 95.3)).

## Results

Baseline characteristics and comparison between the three groups (Healthy Subjects, COVID-19 patients, ILD patients) are presented in Table 1.

**Table 1:** Baseline characteristics of the 3 groups, and comparison of their respective features

Baseline characteristics				P values		
	HS^a^ (N = 70)	COVID19 (N = 83)	ILD (N = 31)	HS vs COVID19	HS vs ILD	ILD vs COVID19
Gender, M (%)	35 (50%)	52 (62.6%)	23(72%)	> 0.05	< 0.05	> 0.05
Age	58 (52–64)	72 (58–82)	69 (62–75)	< 0.001	> 0.05	< 0.01
Leukocytes (/ml)	6.21 (5.13–7.43)	6.3 (4.68–8.61)	8.77 (6.1–11.53)	> 0.05	< 0.01	< 0.01
Neutrophils (/mm^3^)	3.38 (2.81–4.22)	4.65 (3.32–7.21)	5.78 (4.31–8.19)	< 0.001	< 0.001	> 0.05
Lymphocytes (/mm^3^)	2.2 (1.71–2.49)	0.92 (0.65–1.23)	1.97 (0.87–2.2)	< 0.001	> 0.05	< 0.001
CRP (mg/L)	1.0 (0.5–2.4)	60 (27–146)	4.8 (2.1–9)	< 0.001	> 0.05	< 0.001
Fibrinogen (g/L)	2.88 (2.56–3.43)	5.24 (4.03–6.23)	3.43 (3.18–4.15)	< 0.001	> 0.05	< 0.01
KL-6 (U/mL)	254 (191–308)	405 (277–592)	897 (550–1885)	< 0.001	< 0.001	< 0.001

Data are expressed in median (IQR, inter quartile range)

Data are analyzed with Kruskall Wallis test and post Hoc: DunnTest and with Fisher’s test for the sex variable

^a^HS (healthy subjects): Complete blood count: N = 62; CRP: N = 52; Fibrinogen: N = 46

**Fig. 1:**
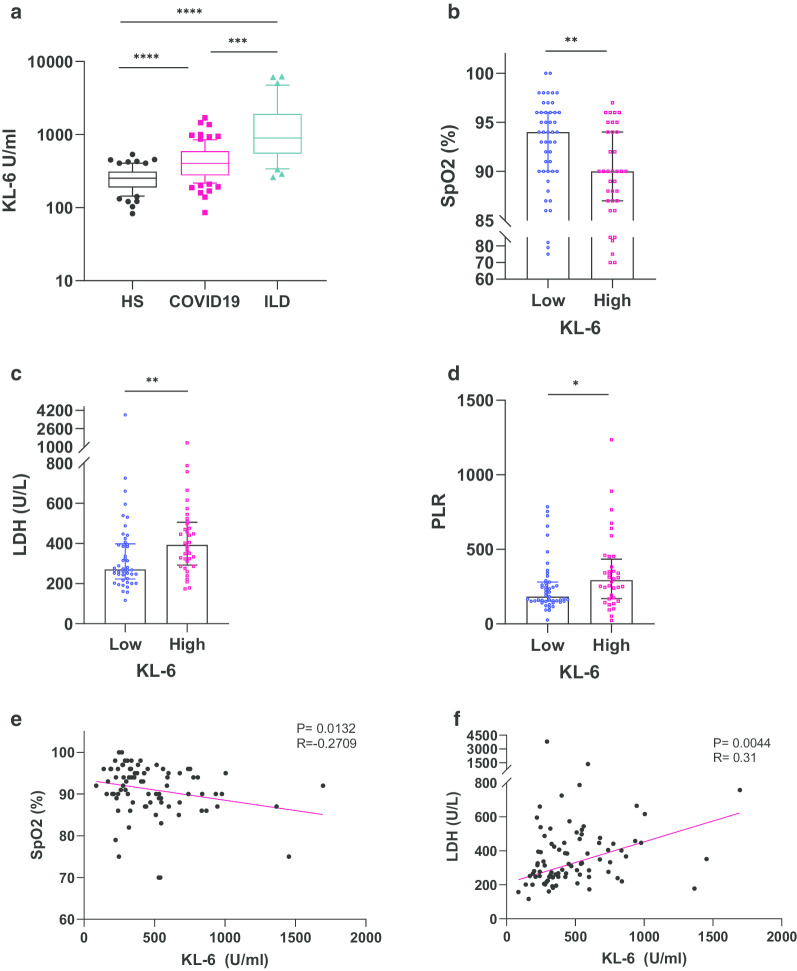
Multi-panel describing results. **a** Comparison of KL-6 levels in healthy subjects, COVID-19 subjects, and ILD-subjects. **b** Comparison of oxygen levels at admission between high and low KL-6 level patients. High KL-6 patients display a significantly lower SpO_2_. **c** Comparison of LDH values between high and low KL-6 level patients. High KL-6 patients display a significantly higher LDH value. **d** Comparison of PLR (platelets/lymphocyte ratio) between high and low KL-6 level patients. High KL-6 patients display a significantly higher PLR. **e** Correlation between KL-6 and oxygen levels. **f** Correlation between KL-6 and LDH levels. Statistics: Data are analyzed by non-parametric unpaired test. **a** Kruskall Wallis test and with post hoc Dunn-test; **b–d**. Mann–Whitney test; **e**, **f**: Spearman correlation*.* *P < 0.05; **P < 0.001; ***P < 0.0001. NB: High KL-6 are KL-6 levels for which values are above mean + 2SD

Our results (Fig. 1) showed that KL-6 levels in COVID-19 patients were increased compared to healthy subjects, but to a lesser extent than in patients suffering from ILD (Fig. 1a). Of interest, increased levels of KL-6 in COVID-19 patients were associated with a more severe lung disease based on oxygen levels at admission to the ambient air [median SpO_2_ of 90% in high KL-6 level patients (n = 36) versus 94% in low KL-6 level patients (n = 47)]; p = 0.013; r = − 0.271, Fig. 1b, e). However, high KL-6 were not linked to severe dyspnea (p = 0.585), or to ICU admission (p = 0.434). Similarly there was no association between high KL-6 levels and mortality (p > 0.05). Concerning laboratory values, despite an increase in CRP and fibrinogen levels in COVID-19 patients, there was no correlation between high KL6 levels and CRP (p = 0.482) or fibrinogen (p = 0.288). Confirmatory to previous results focusing on biological markers associated with severe COVID-19 infection, high KL-6 was correlated with high LDH levels (r = 0.31 p = 0.004, Fig. 1c, f). Concerning platelet/lymphocyte ratio (PLR), we did not find a global correlation with KL-6, but noteworthy, high-KL-6 levels were associated to higher values of PLR (p = 0.04, Fig. 1d).

## Discussion

KL-6 is known to be linked to alveolar damage [7]. Previous studies have demonstrated that COVID-19 infections were associated to potential lung fibrosing process induced by alveolar damage [8]. Therefore, using biomarkers in order to predict lung evolution of severe COVID-19 infections could be of interest in order to identify patients with high risk of experiencing severe lung disease as well as significant parenchymal sequelae.

Our study confirms that KL-6 level could be an indicator of COVID-19 infection severity. This was verified by the parallel impact on oxygen levels. Nevertheless, high KL-6 levels were not associated to more pronounced dyspnea, as this clinical feature was largely encountered in COVID-19 patients, regardless of their KL-6 levels. Similarly, there was no link between high KL-6 levels and ICU admission or death, for which we assume many other factors ought to be considered. Still, high KL-6 levels were interestingly correlated with other indicators of disease severity such as high LDH and PLR, as already mentioned in previous studies [9, 10].

Most recent studies focus on the current clinical-laboratory or CT features of COVID-19, but only a few are questioning the long-term impact of such infection. However, tissue inflammation and subsequent healing of the lungs can lead to pulmonary fibrosis. Our study confirms that KL-6—a recognized marker in lung fibrosing process—is increased in COVID-19 patients. This finding is in line with recent studies, describing the rise of other fibrosis biomarkers in this infection [8]. Therefore, the hypothesis of KL-6 as an indicator of lung disease (acute hypoxemia), and even of a possible evolution towards a fibrotic process in the course of a COVID 19 infection must be considered.

## Conclusion

Taken together, these results suggest that KL-6 could be a good biomarker to assess ILD severity in COVID-19 infection. Concerning the therapeutic response and prognosis prediction, more studies are necessary.

## Data Availability

The datasets used and/or analysed during the current study are available from the corresponding author on reasonable request.

## Abbreviations

HS: Healthy subjects
ICU: Intensive care unit
ILD: Interstitial lung disease
KL-6: Krebs von den Lungen 6
LDH: Lactate deshydrogenase
PCR: Polymerase chain reaction
PLR: Platelet/lymphocyte ratio
SARS-COV 2: Severe acute respiratory syndrome-coronavirus
